# The Numerical Analysis of Replenishment of Hydrogel Void Space Concrete Using Hydrogels Containing Nano-Silica Particles through ELM-ANFIS

**DOI:** 10.3390/gels8050299

**Published:** 2022-05-13

**Authors:** Ji Min, Yousef Zandi, Alireza Sadighi Agdas, Ali Majdi, H. Elhosiny Ali, Amin Jan, Anas A. Salameh, Ahmed Abdel Khalek Ebid

**Affiliations:** 1 Chongqing Vocational Institute of Engineering, Chongqing 650224, China; eden-jimin@foxmail.com; 2 Department of Civil Engineering, Tabriz Branch, Islamic Azad University, Tabriz 51579, Iran; 3 Ghateh Gostar Novin Company, Tabriz 51579, Iran; alireza.sadighi.agdas@hotmail.com; 4 Department of Building and Construction Techniques, Al-Mustaqbal University College, Hillah 51001, Iraq; alimajdi@mustaqbal-college.edu.iq; 5 Advanced Functional Materials & Optoelectronic Laboratory (AFMOL), Department of Physics, Faculty of Science, King Khalid University, Abha 61413, Saudi Arabia; hibrahim@kku.edu.sa; 6 Research Center for Advanced Materials Science (RCAMS), King Khalid University, Abha 61413, Saudi Arabia; 7 Physics Department, Faculty of Science, Zagazig University, Zagazig 44519, Egypt; 8 Faculty of Hospitality, Tourism and Wellness, Universiti Malaysia, City Campus, Kota Bharu 16100, Kelantan, Malaysia; aminjan@umk.edu.my; 9 Department of Management Information Systems, College of Business Administration, Prince Sattam Bin Abdulaziz University, Al-Kharj 11942, Saudi Arabia; a.salameh@psau.edu.sa; 10 Structural Engineering and Construction Management, Faculty of Engineering, Future University, Cairo 11835, Egypt; ahmed.abdelkhaleq@fue.edu.eg

**Keywords:** hydrogel, void space, concrete, nano-silica, ELM-ANFIS

## Abstract

Currently, Nano-materials are gaining popularity in the building industry due to their high performance in terms of sustainability and smart functionality. In order to reduce cement production and CO_2_ emissions, nano-silica (NS) has been frequently utilized as a cement alternative and concrete addition. The influence of Nano-silica-containing hydrogels on the mechanical strength, electrical resistivity, and autogenous shrinkage of cement pastes was investigated. The goal of this study was to identify the main structure–property relationships of water-swollen polymer hydrogel particles used as internal curing agents in cementitious admixtures, as well as to report a unique synthesis process to combine pozzolanic materials with hydrogel particles and determine the replenishment of hydrogel void space. Experiments were designed to measure the absorption capacity and kinetics of hydrogel particles immersed in pure water and cementitious pore solution, as well as to precisely analyze the data derived from the tests using hybridized soft computing models such as Extreme learning machine (ELM) and Adaptive neuro-fuzzy inference system (ANFIS). The models were developed, and the findings were measured using regression indices (RMSE and R2). The findings indicated that combining nano-silica with polymeric hydrogel particles creates a favorable environment for the pozzolanic reaction to occur, and that nano-silica assists in the refilling of hydrogel void space with hydrated cement phases.

## 1. Introduction

In response to an increasing need for concrete that is more desirable in terms of durability as well as strength, high-performance concrete (abbreviated as HPC) was created with a low carbon footprint and a high level of durability and strength while keeping a low carbon footprint [1,2,3,4,5]. Thus, because of the low water-to-cement ratio (w/c) used in its construction, HPC is susceptible to shrinkage along with self-desiccation [6,7,8,9,10]. As previously mentioned, SAP particles (superabsorbent polymer) are employed to tackle this problem and have shown promising results as internal curing agents [11] as shown by past research. SAPs have the ability to absorb a large amount of water during the mixing process and then expel it as concrete matures, aiding the hydration reaction of concrete [12,13,14,15,16,17,18]. SAPs are beneficial for the prevention of self-desiccation [19], the reduction of autogenous shrinkage, the securing of fractures [20], the refinement of resistance to freeze thaw attacks, and assistance of microstructure [21] as a result of increased hydration, all of which improve the durability and strength of concrete [22,23,24,25,26,27]. However, it should be emphasized that the installation of SAPs in concrete is fraught with difficulties. First and foremost, to guarantee that mechanical strength of bulk concrete is not adversely affected by huge gaps formed due to dehydrated SAPs, size and dosing of hydrogel-based particles must be strictly regulated. Dehydrated SAPs are a type of additive that is used to increase the mechanical strength of concrete [28,29,30,31,32,33]. The chemical structures of SAPs also impact the rheological qualities of freshly mixed mortar [34], as previously stated. Following that, the time of water discharge is crucial because in the course of the acceleration stage, desorption of the cement paste supports the enhancement of hydration and reduction of autogenous shrinkage. Furthermore, it is vital to realize that SAPs are not inert chemical substances when they are used [35,36,37,38]. Among the SAPs employed in cementitious systems, poly (acrylic acid-acrylamide) constitutes the vast majority [39,40]. Several studies have established that multivalent cations produce fast dehydration (or the deswelling) of bulging hydrogel particles, and in basic pore solutions of newly combined concrete multivalent cations are plenteous [35,36,41,42,43]. When exposed to alkaline circumstances, carboxylic acid groups in acrylic acid (sodium acrylate) polymer networks completely collapse [44,45,46,47,48]. This is due to the fact that in these networks, the carboxylic acid groups turn anionic and can connect with free cations [49,50,51,52,53]. As a consequence, SAPs that have acrylic acid are more inclined than other SAPs in releasing water prematurely within cementitious environments, increasing effective water content and decreasing mortar compressive strength [54,55,56,57,58,59]. According to previous research, the desorption of SAPs that are rich in acrylic acid occurs within minutes after free swelling trials, limiting the success of the internal cure if it takes place in the midst of the latent period [60,61,62,63,64]. On account of the acrylic acid’s sensitivity to the concentration of pore solution in cementitious mixes, this study investigated hydrogel particles that are acrylamide-based but not significantly ionized in basic surroundings, which should have more favorable and stable swelling behavior in cementitious mixes [65,66,67,68]. Several studies have shown that acrylamide-rich SAPs have greater immunity to alterations in pore-solution chemistry [69] and that within the gaps in the cement microstructure formed following SAP dehydration, inorganic phase formation occurs [70,71,72,73,74,75]. In the case of hydrogel particles containing just acrylamide, this “void-filling” effect was shown to be much more pronounced [76,77,78,79,80]. It was also discovered that increasing the concentration of acrylamide in the hydrogel helped to increase cement matrix adhesion, which seemed to accelerate hydrogel particle desorption as a result of capillary effects [81,82,83,84,85].

The durability difficulties associated with autogenous shrinkage cracking in this material system as a result of the low w/c ratio utilized for fabrication of high-performance concrete [86] are a significant impediment to the widespread application of high-performance concrete [87,88,89,90,91,92]. Reduced relative humidity induces capillary forces in the material microstructure as a consequence of self-desiccation [93,94,95,96,97]. When a material develops cracks, the pace at which hazardous components are transferred into the substance rises substantially, resulting in physical and chemical deterioration [98,99,100,101,102]. To alleviate autogenous shrinkage-induced cracking and the related durability problems [103], internal curing agents such as expanded clay, saturated lightweight aggregates, pumice, and superabsorbent hydrogels have been employed in past [104,105,106,107,108,109]. It has been established that superabsorbent hydrogels may decrease autogenous shrinkage [11], minimize fracture formation [110], and enhance freeze–thaw resistance in addition to other properties. Furthermore, earlier research has shown [111] that hydrogels have the potential to self-heal after being damaged. However, the use of hydrogels in cementitious materials has a drawback in terms of mechanical strength since the hydrogels create considerable voids inside the material which makes the material less durable.

In order to understand how hydrogels affect the characteristics of cementitious materials, it is vital to understand the chemical characteristics, physical characteristics, and combined design of cementitious materials of hydrogels [34]. The most commonly encountered hydrogels in cementitious mixes are cross-linked polymers of acrylic acid salts or copolymers of acrylic acid salts and acrylamide [112,113,114,115,116]. In addition, pH and ionic strength of solution have an effect on the behavior of polymer networks of the hydrogels since they are ionizable. It is possible to leverage this characteristic of hydrogels in order to customize them in accordance with the chemistry of cementitious substances in order to acquire the expected effect. Nanosilica (NSi), silica fume, metakaolin, and fly ash are pozzolanic additives that are employed in the creation of high-performance ceramics to extend the service life and durability of the material [117,118,119,120,121,122]. Hydrogels have been examined before in relation to their influence on the activity of cement blends, including fly ash, silica fume, ground glass, and ground-granulated blast furnace slag [123,124,125,126,127]. When compared with various pozzolans, amorphous NSi has shown stronger pozzolanic reactivity as a result of its large specific surface area [128,129,130,131,132]. Amorphous silica was found to be present in large amounts in a prior study on application of cementitious materials with a mix of internal curing and pozzolanicity. It has also been attempted in the past to make hydrogels from fly ash and rice husk ash as well as for soil-conditioning uses and oil recovery. Additionally, NSi has been incorporated into several composite systems for oil-recovery uses [133].

**Figure 1:**
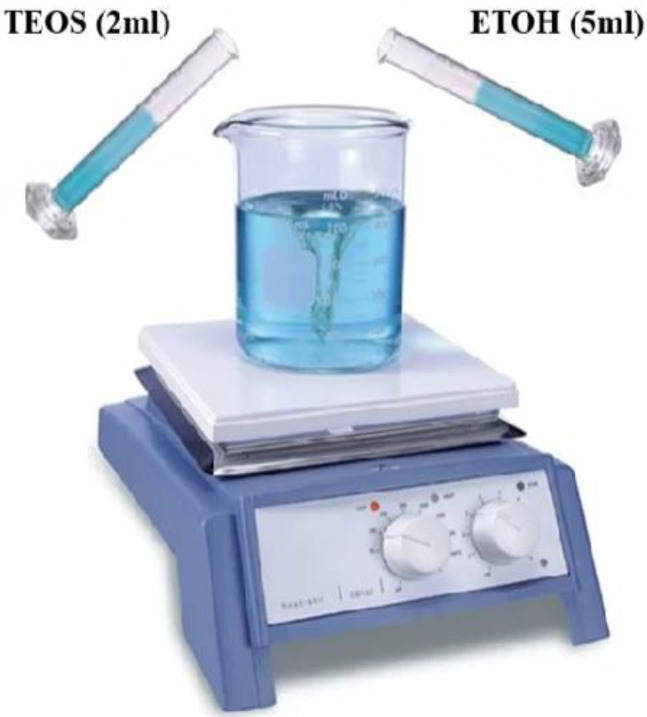
Synthesis method of SIO_2_ nanoparticles.

Amorphous NSi, in particular, due to its high specific surface area, has shown increased pozzolanic reactivity compared with other pozzolans. Prior studies examined the use of materials with a combination of pozzolanicity and internal curing in cementitious materials. In those studies, the internal curing material consisted of a porous material with high amorphous silica content. Figure 1 shows the synthesis of SIO_2_ nanoparticles. The use of hydrogels containing rice husk ash in oil recovery and fly ash in soil-conditioning applications were also attempted in the past. NSi has also been used in other composite systems in oil-recovery applications [133,134,135,136,137].

The microstructure of high-performance concrete (HPC) is highly thick when compared with conventional concrete due to a much lower w/c, implying that the water in the mix is used throughout the hydration process. The dense microstructure results in a construction that is both durable and long-lasting while also having a low environmental impact. One of the most challenging technical issues associated with using high-performance composites (HPC) is autogenous shrinkage, which happens during the curing process and ultimately results in fracture development, increased porosity, and a loss in overall strength [138,139,140,141,142]. A lack of water penetration due to the thick microstructure of HPC means that the standard external curing processes employed in the building industry are not able to completely avoid autogenous shrinkage [143,144,145,146]. Because of this, HPC internal curing delivers a greater amount of water for processes related to hydration from inside the concrete [110], resulting in a reduction of shrinkage [147,148,149,150,151]. This is a good process of internal curing [152] because superabsorbent polymer hydrogel particles are able to collect and discharge a lot of water, which is necessary to drive hydration processes [103,153,154,155,156]. In vitro studies have demonstrated that internal curing using hydrogel particles may decrease autogenous shrinkage, fracture creation, improve autogenous sealing capacity [157], enhance freeze–thaw cycle resistance, and improve longevity. Silica fume (SF), fly ash, and other similar minerals are pozzolanic substances often used in the production of high-performance ceramics [158,159,160,161,162,163].

**Figure 2:**
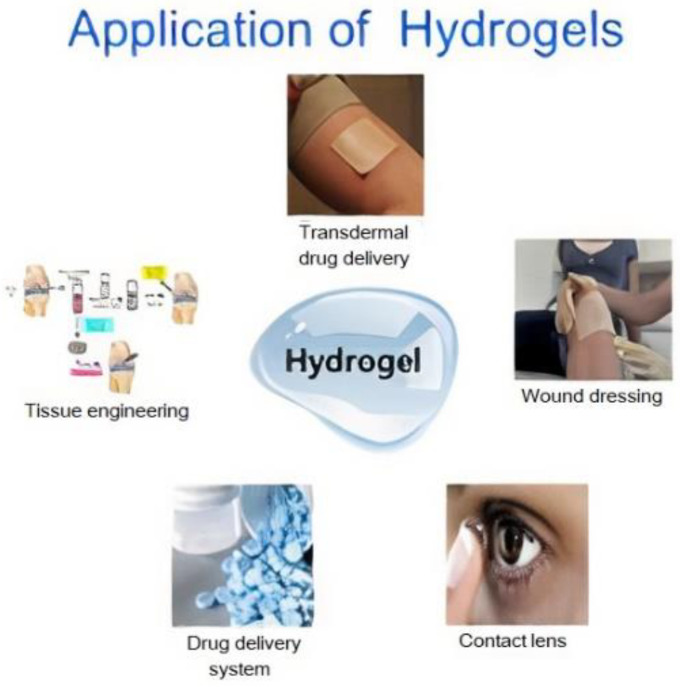
Applications of hydrogel.

Due to the arrival of nanotechnology, many types of amorphous nano-silicas with a large particular surface area are utilized since it has been revealed that in comparison to traditional SF, they have greater pozzolanic activity [164,165,166,167,168]. A pozzolan with internal curing, expanded shale, and porous rice husk ash has been employed successfully in studies. When fine aggregates or cement were not available, porous substances with inherent pozzolanic characteristics (i.e., a high fraction of amorphous silica phases) were used in their place. Though polyacrylate-based hydrogels with fly ash are developed to be used for improved oil recovery and hydrogels with rice-husk ash are used in soil conditioning, there have been no previous attempts to incorporate nano-silica directly into a hydrogel particle for use in internally cured cement-based materials. In this research, we describe a straightforward synthetic approach for combining nano-silica (SiO_2_) particles with a polymeric internal curing agent to form a composite material (hydrogel) (Figure 2). In the case of nano-silica-containing compositions, the dosage was determined to be 8.5 percent SiO_2_ by monomer weight. The efficiency of these hydrogel particles was determined by the use of cement pore solution and gravimetric swelling tests in reverse osmosis (RO) water, respectively. Additionally, the uses of backscattered electron microscopy to determine the influence of the hydrogel inclusion on the space structure and the creation of a hydrated phase in cement pastes after hydrogel particles were integrated. It has been observed that hydrogel particles that are employed as internal curing agents have an impact on the cement chemistry, namely: production of calcium–silicate–hydrate (CSH) and calcium hydroxide (CH) phases [169]. We recently observed that hydrogel particles comprised mostly of polyacrylamide are capable of producing large quantities of CH phases inside hydrogel void space [21], which was previously unknown. As a consequence, the mixing of pozzolanic material into a hydrogel network may result in the creation of an additional CSH phase, hence improving the mechanical properties and extending the lifespan of internally cured concrete. According to this research on hydrogel–ion interactions and how hydrogel chemistry influences cement paste microstructure, it may be possible to modify the chemical structure of hydrogel particles in order to boost the amount of calcium hydroxide (CH) phases that develop inside the hydrogel void. According to current expectations, the combining of nano-silica in water-soluble hydrogel particles would allow for the development of additional CSH in the empty space by employing a combination of water-soluble hydrogel particles and pozzolan and inside the hydrogel.

### Objectives and Problem Statements

**Figure 3:**
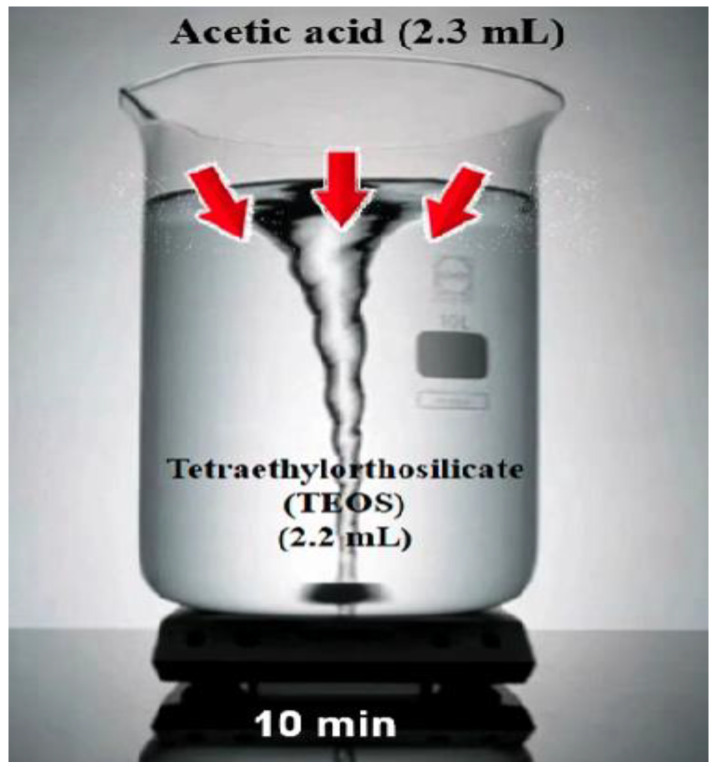
Adding 2.3 mL acetic acid to 2.2 mL Tetraethlorthosilicate (TEOS) and stirring for 10 min.

**Figure 4:**
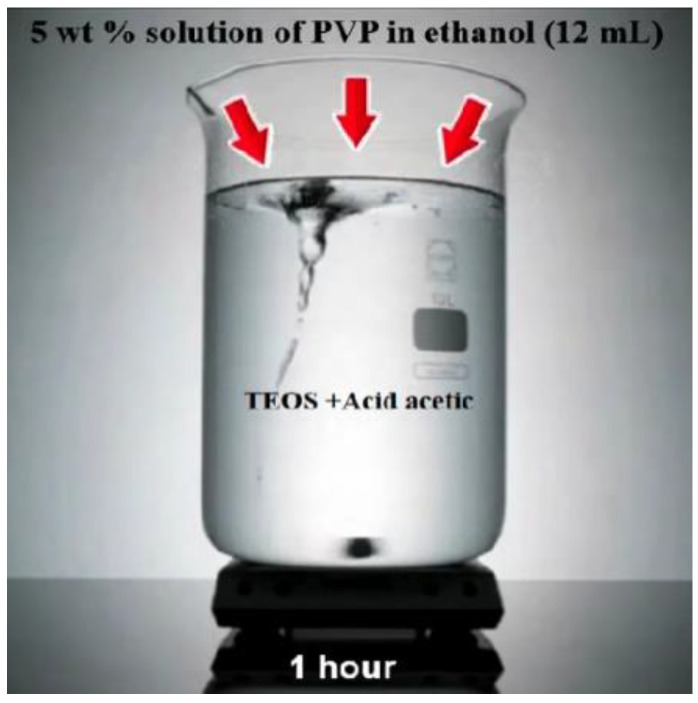
Adding 5 wt% solution of PVP.

Presently, hydrogels have received a lot of interest as a self-healing and internal curing agent. Hydrogel’s size distribution and absorption/desorption capabilities are both versatile, allowing it to be modified to particular mix designs. The first goal of this study was to see how the chemical structure of hydrogels affects their activity in cementitious materials and how to fill hydrogel void spaces in cement by the use of Nano-silica particles. Understanding how hydrogel activity, notably absorption and desorption in cementitious materials, affects the microstructure and characteristics of cementitious materials requires an expertise on hydrogel behavior. Further than the well-known chemical interactions between the pore solution and hydrogels, this study intends to fill a gap of knowledge in comprehending the variables influencing hydrogel absorption in cement mixes. The outcomes of the empirical tests are then examined using ELM-ANFIS. Figure 3 shows the addition of 2.3 mL acetic acid to 2.2 mL Tetraethlorthosilicate (TEOS) and stirring for 10 min and Figure 4 shows the addition of 5 wt% solution of PVP.

## 2. Materials and Methods

### 2.1. Materials

Free radical polymerization was used to create the cross-linked polyacrylamide hydrogels that were used in this investigation. Acrylicamide monomers (AM) were combined into distilled water containing sodium silicate to produce varied compositions of NSi/AM equal to 0 percent, 10 percent, and 20 percent, respectively. *N*,*N*′-methylenebisacrylamide (MBA) and ammonium persulfate were added to the solution to act as an initiator and a cross-linking agent, respectively. The solution was poured into the beaker, which was then placed in an oven at 60 degrees Celsius for three hours until it gelled. It was necessary to soak the hydrogels in distilled water for one day in order to eliminate the monomers that did not react with one another before drying at 80 degrees Celsius. The hydrogels were dried and crushed in a coffee grinder, after which they were sieved to produce a powder with a particle size range of 75–425 m. The results were published in the journal Biomaterials. Scanning electron images of the hydrogel powders taken at different magnifications are shown in Figure 1.

### 2.2. Silica Additive Materials Incorporated into Hydrogel

Other additives can be put into hydrogels to improve cementitious healing qualities. Three distinct materials, Colloidal silica (CNSi), Water Glass (WG) and Nano-silica particles (NSi), and Water Glass were introduced into hydrogels in this research.

### 2.3. Nano-Silica Particles (NSi) and Colloidal Silica (CNSi)

**Figure 5:**
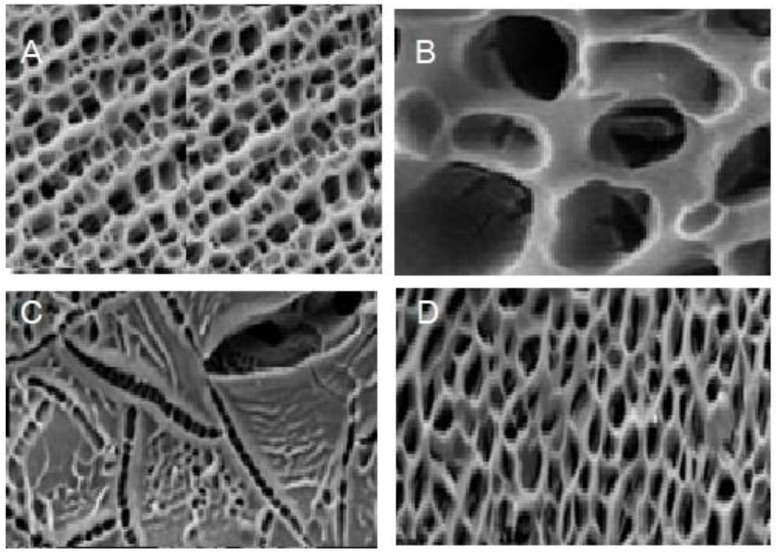
SEM images of lyophilized (**A**) DMAPM AAm hydrogel and (**B**) DMAPMAAm/nano-SiO_2_, (**C**) DMAPMAAm/amine-modified hydrophilic nano-SiO_2_, and (**D**) DMAPMAAm/EP nanocomposite hydrogels.

Silicon dioxide nanoparticles, sometimes referred to as nano-silica or silica nanoparticles, are a type of nano-reinforcement that can be thought of as a smaller, manufactured version of silica fume. Nano-silica is available in both solid and colloid forms, although colloidal nano-silica is preferable due to collection in the solid form (Figure 5). The inclusion of nano-silica in the hydrogel was inspired by its favorable effects on the cementitious materials’ microstructure, mechanical characteristics, and hydration. It is one of the most widely used admixtures in the concrete sector as a result of its small size, void-filling capabilities and pozzolanic activity. Various amounts of nano-silica, ranging from 1 to 4 percent, have been used in many investigations and tests. The results reveal an improvement in mechanical characteristics as well as a reduction in pore volume. Since it has the function of an activator to boost pozzolanic reaction, a small amount of nano-silica increases compressive strength dramatically. Although the granularity of nano-silica increases the initial concrete strength, the final strength of concrete produced using coarse nano-silica was shown to be higher. For both cases, the optimal Nano-silica dosage was between 1.0 and 1.5 percent. Likewise, combining Nano-silica and steel fibers significantly improves flexural strength. The addition of 1.5 percent nano-silica to HPC improves its flexural strength by roughly 15%.

A more opaque appearance was seen in hydrogel powders containing NSi compared with hydrogel powders that did not include NSi, and the opaqueness was enhanced as the quantity of NSi present in hydrogels increased. This substance is meant to be physically bonded and maintained inside the polymer networks of hydrogels. In this study, nano-silica particles were exposed to X-ray diffraction (XRD) using a Siemens D500 diffractometer (30 mA, 50 kV) with a scanning rate of 0.02°/s in the (10°–40°) range and a scanning rate of 0.02°/s (Siemens AG, Berlin and Munich, Germany). Using a top-loaded metal sample container that was forced against a paper surface, the nanoparticles were softly packed into the specimen in order to minimize any preferred orientation. It was discovered that when nano-silica particles are suspended in DI water, their zeta potential can be measured using a Nanosizer Nano-z instrument (Malvern Instruments, Malvern, United Kingdom). Two samples were created at different pH values, i.e., 6.3 0.1 and 12.4 0.1, by the gradual incorporation of a 2 M NaOH solution. The pH values of the samples were measured using an electronic pH meter. Then, 30 g/mL concentration (1 weight percent) of nanoparticles was mixed into the solution before testing, and it was allowed to equilibrate at 25 °C for two hours before running the experiment. This phase allowed bigger particle aggregates to settle out of the solution, leaving behind a suspension of scattered smaller aggregates and isolated particles (less than a few microns in size) that could be analyzed later on in the process. There were three measurements taken from each sample, and an average was calculated.

### 2.4. Compressive Strength Measurements

**Figure 6:**
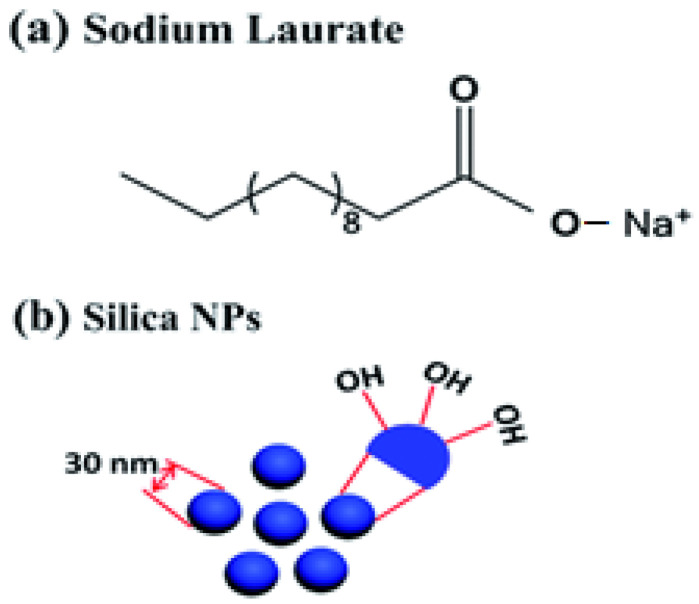
Hydrogel formed by the co-assembly of sodium laurate and silica nanoparticles.

Compressive strength tests on cement paste samples aged 3, 7, and 28 days were carried out using an Insight 820.300-SL machine with a load capacity of 300 kN at a constant strain rate of 1 mm/min (MTS Systems Corp., Eden Prairie, MN, USA). For every sample, 3 specimens were analyzed, and mean compressive strength as well as standard deviation were computed for each specimen. Figure 6 shows hydrogel formed by the co-assembly of sodium laurate and silica nanoparticles.

### 2.5. Hydrogel Absorption Results

**Figure 7:**
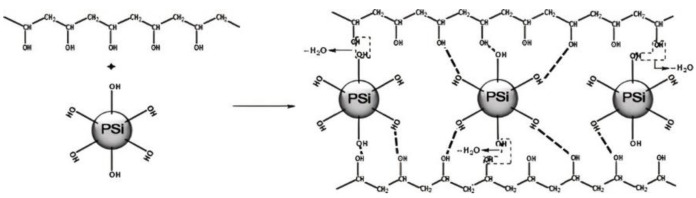
Structure and property of polyvinyl alcohol/precipitated silica composite hydrogels for microorganism immobilization.

Swelling capabilities of hydrogel particle samples within the pore solution as well as RO water are discussed in detail in this section. The addition of NS to pure (silica-free) AM particles resulted in increased absorption in RO water at a maintained crosslink density of 2 percent with absorption increasing by 19 percent and 55 percent, respectively, at equilibrium (24 h) for NS doses of 1 percent and 10 percent. When SF doses of 1 percent and 10 percent were used in conjunction with equilibrium absorption, the results showed a little improvement in equilibrium absorption of 2 and 20 percent, respectively. Aside from that, lowering crosslink density for pure (silica-free) particles increased swelling capacity by 110 percent, and a similar trend was seen for silica-containing particles. For example, when NS-10-0.5 was contrasted against NS-10-2, it was shown to have 80 percent larger equilibrium swelling capacity than the latter. Since the naturally present pore solution ions lowered the osmotic driving force for water absorption, the absorption capabilities for every hydrogel sample was lower in the pore solution. It is worth noting that no silica particle remains were detected in the beakers following swelling trials, showing that the SF and NS particles were physically contained inside the hydrogel particles even when the swelling was at its maximum. Figure 7 shows the structure and property of polyvinyl alcohol/precipitated silica composite hydrogels for microorganism immobilization.

### 2.6. Extreme Learning Machine (ELM)

Artificial Intelligence (AI), as a novel approach, has been developed widely in various fields [170,171,172,173,174] and, compared with other numerical methods [175,176,177,178,179], has several advantages such as being more time-saving and accurate [180,181,182,183,184]. The capability of AI algorithms in predicting reliable results has been shown in recent years, which has led to the development of these types of techniques [185,186,187,188,189]. Extreme learning machine (ELM) [190], a recently introduced fast-learning neural algorithm for SLFNs, was newly created to enhance the performance of SLFNs [191,192,193,194,195]. In contrast to traditional neural network learning algorithms such as BP algorithms that have difficulty manually tuning control parameters like learning epochs, learning rate, and so on [196,197,198], and/or local minima, ELM is completely automated without the need for repeated tuning and, theoretically, does not require human participation at any point in the process [199,200,201,202,203]. Furthermore, as compared with other traditional techniques [204,205,206,207,208], the learning pace of ELM is much faster. Hidden node learning factors such as biases and input weights may be randomly given separately in the ELM technique, and the network’s output weights can be derived systematically by performing a generalized inverse operation on the network’s weights [209,210,211,212,213]. A fixed nonlinear transformation may be used to efficiently close up the training phase without the need for a time-consuming learning procedure to be performed [214,215,216,217]. A last point to mention is that the ELM approach contains an outstanding generalization performance. Furthermore, it has been demonstrated that the normal ELM has universal estimation ability when using RBF activation functions or additive [218] functions. Several real-world problems, including regression and classification, have been successfully addressed using ELM [219,220,221]. The creation of an ELM model includes many processes including the construction of the SLFN, random selection of the network’s biases and weights, and the computation of output weights via inversion of the hidden layer output matrix among others. One SLFN with *L* hidden nodes is theoretically investigated for a dataset with m-dimensional target vectors, *N* training samples, and n-dimensional input vectors. The dataset is modeled as follows:

(1) $$\sum_{i=1}^{L}\;\beta_{i}G\left(w_{i}\cdot x_{j}+b_{i}\right)=o_{j}\;j=1,\;2,\;3,\;\ldots ,\;N$$

G = activation performance

$w_{i}={\left[w_{i1},\;w_{i2},\;\ldots ,\;w_{in}\right]}^{T}$ = weight vector connecting $i^{th}\;input\;neurons$ to hidden neuron

$x_{j}={\left[x_{j1},\;x_{j1},\ldots ,\;x_{jm}\right]}^{T}$= input vector

$\beta_{i}={\left[\beta_{i1},\beta_{i2},\;\ldots ,\;\beta_{im}\right]}^{T}$ = weight vector connecting output neurons to hidden neurons

$b_{i}={\left[b_{i1},b_{i2},\;\ldots ,\;b_{im}\right]}^{T}$ = bias vector

$o_{j}={\left[o_{j1},o_{j1},\;\ldots ,\;o_{jm}\right]}^{T}$= output vector

Assuming that one SLFNN with activation function G and L hidden neurons could give the targets ($t_{j})$ with 0 error, e.g., $\sum_{j=1}^{L}\;\|o_{j}-t_{j}\|=0$, Equation (1) could be as Equation (2):

(2) $$\sum_{i=1}^{L}\;\beta_{i}G\left(w_{i}\cdot x_{j}+b_{i}\right)=t_{j}\;\;\;\;j=1,\;2,\;3,\;\ldots ,\;N$$

$={\left[t_{j1},t_{j2},\;\ldots ,\;t_{jm}\right]}^{T}$ = target vector

Additionally, this N equation could be compactly communicated as $t_{j}$

(3) Hβ=T

(4) $$H={\left[G\left(w_{1}+x_{1}+b_{1}\right)\;\ldots \;G\left(w_{L}\cdot x_{1}+b_{L}\right)\;\vdots \;\ldots \;\vdots \;G\left(w_{1}+x_{N}+b_{1}\right)\;\ldots \;G\left(w_{L}\cdot x_{N}+b_{L}\right)\;\right]}_{N\times L}$$

and

(5) $$\beta ={\left[\beta_{1}^{T}\;\vdots \;\beta_{L}^{T}\;\right]}_{L\times m}\;\mathrm{and}\;T={\left[t_{1}^{T}\;\vdots \;t_{N}^{T}\;\right]}_{N\times m}$$

If minimal difference among the right side (target variables) and left side (predicted variables) of Equation (6) occurs, output weights are acquired, i.e., min ‖Hβ−T‖. It was also discovered that when the output weight is set to the following, the least error between the predicted and target variables occurs:

(6) $$\hat{\beta }=H^{†}T$$

$$\hat{\beta }=Output\;weight\;vector$$

$$H^{†}=Moor-Penrose\;generalized\;inverse\;matrix$$

T=Target vector

**Figure 8:**
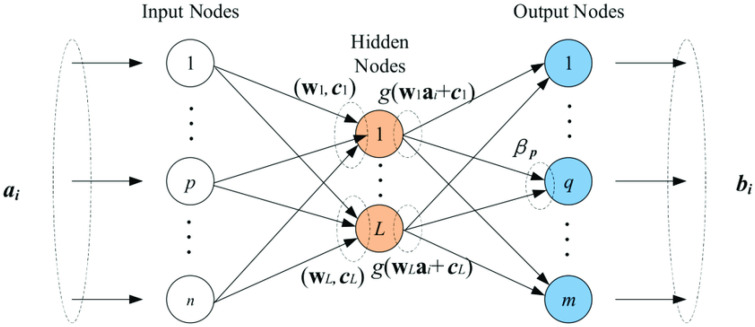
ELM diagram.

Because ELM lacks an optimization approach, human inferences and training time are considerably reduced (Figure 8).

### 2.7. Adaptive Neuro-Fuzzy Inference System (ANFIS)

**Figure 9:**
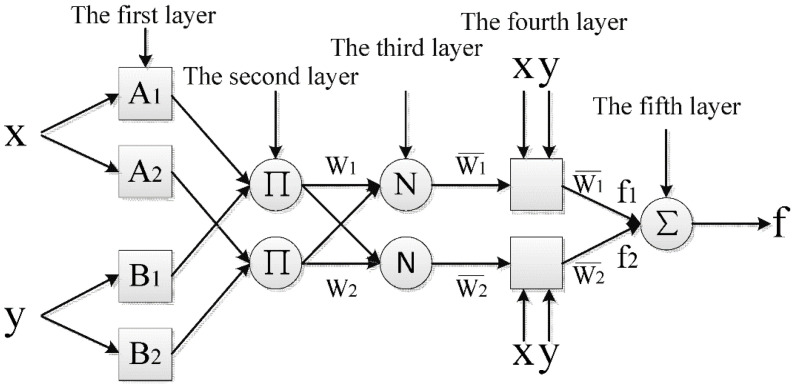
ANFIS diagram.

As previously said, ANFIS is a multilayer feed-forward network consisting of nodes that are linked by direct connections and every node that acts on its receiving signals in a predefined way. Direction of signals from one node to another node is described by each connection in an adaptive network, and as a result, each link has no weight(s). With the present parameters established in mind, ANFIS generates a fuzzy-inference system (FIS) (Figure 9) that is based on the input/output nature of data with membership functions modified using either a gradient decent technique or in conjunction with the least-squares approach. ANFIS also employs a learning algorithm to precisely discover the ideal settings for FIS parameters that are comparable to one another. During the training phase, the parameters are fine-tuned to ensure that the disparity among observed and predicted values is as little as feasible. An ANFIS layer structure is comprised of five layers, each of which has its own name. The core of ANFIS is a fuzzy-inference system (FIS). It is the initial layer that accepts input (crisp) values (x and y) and converts them to fuzzy values via the application of membership functions (MFs). In the ANFIS knowledge base’s rule base, there are two fuzzy IF-THEN rules of the sort developed by Sugeno and Takagi:

Rule 1: if x is A_1_ and y is B_1_, then f_1_ = p_1_ x + q_1_ y + r_1_

Rule 2: if x is A_2_ and y is B_2_, then f_2_ = p_2_ x + q_2_ y + r_2_

Each node of the first layer is selected as an adaptive node with a node function $O_{i}$,

(7) $$O_{i}{}^{1}=\mu A_{i}(x)$$

where:

$A_{i}$ = a linguistic label

μ = membership function

In FIS development, the bell-shaped membership function is often utilized because of its enhanced capacity in the regression of nonlinear data. Described below is a bell-shaped membership function with a maximal value of 1 and a minimal value of 0. It has the following characteristics:

(8) $$\mu (x)=bell(x;a_{i},b_{i},c_{i})=\frac{1}{1+{\left[{\left(\frac{x-c_{i}}{a_{i}}\right)}^{2}\right]}^{b_{i}}}$$

where:

$\left\{a_{i},b_{i},c_{i}\right\}$ = premise parameters

x = input

It is the second layer that amplifies the incoming signals and then transmits the result to the next tier of the layers. For example:

(9) $$w_{i}=\mu A_{i}(x)\times \mu B_{i}(x)\;i=1,\;2$$

Each node’s output reflects the strength with which a rule is being fired.

The rule layer (the third layer) calculates the ratio of the node firing strength of the rule to the node firing strength of the other nodes using the following formula:

(10) $$w_{i}{}^{*}=\frac{w_{i}}{w_{1}+w_{2}}\;i=1,\;2$$

The outputs $w_{i}{}^{*}$ are denoted as *normalized firing strength.*

Each node in the defuzzification layer (the fourth layer) performs one of the following node functions:

(11) $$O_{i}{}^{4}=w_{i}{}^{*}f_{i}=w_{i}{}^{*}\left(p_{i}x+q_{i}y+r_{i}\right)$$

where:

$w_{i}{}^{*}$ = the output of the third layer

$\left\{p_{i},q_{i},r_{i}\right\}$ = *consequent parameters*

After all the incoming signals are added together at the output layer (which is the fifth layer), the total output is computed:

(12) $$O_{i}{}^{5}=f=\sum_{i}w_{i}{}^{*}f_{i}$$

A threshold value is chosen among the observed and predicted values in this method. The error value is then determined and reduced by the update of the premise value as well as resulting parameters. This technique is repeated until the error falls below the threshold, at which point the initial FIS is trained (Figure 9).

## 3. Result and Discussion

### 3.1. Model Performance Indicators

**Figure 10:**
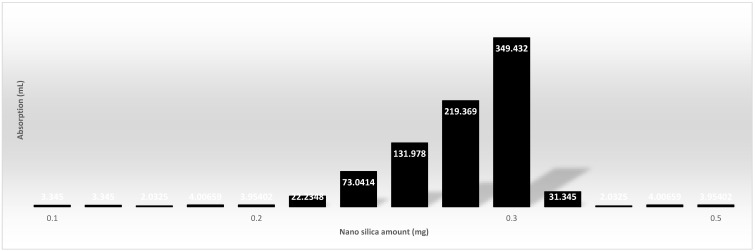
Addition of SF also increased the equilibrium absorption by 2% and 20% for SF dosages of 1% and 10%, respectively.

**Figure 11:**
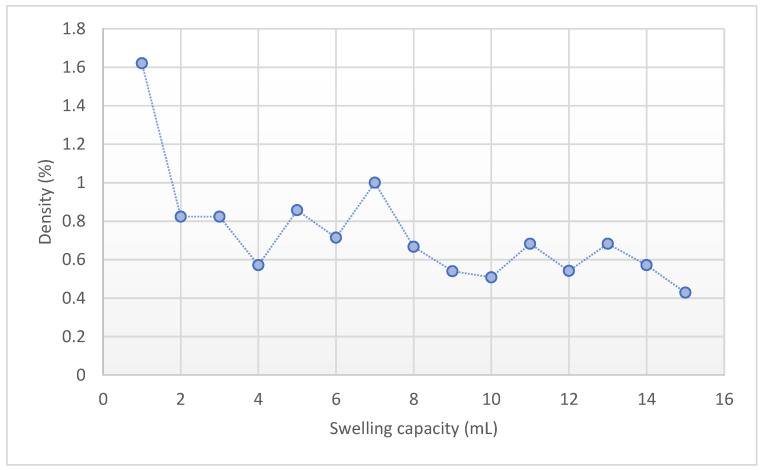
Decrease of density by raise of swelling capacity by 110% for the pure (silica-free) particles.

**Figure 12:**
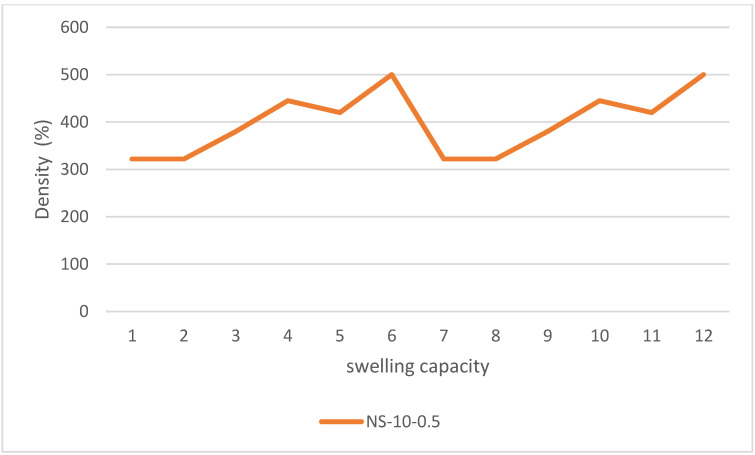
Observed 80% increase in equilibrium swelling capacity of NS-10-0.5 compared with NS-10-2.

According to the derived data, 70% of it was assigned for the training phase and 30% was used for the testing phase. The regression indices of root mean square (*RMSE*), Pearson correlation coefficient (*r*), and determination coefficient (*R*^2^) were applied via MATLAB. Figure 10 indicates that the addition of SF also increased the equilibrium absorption by 2% and 20% for SF dosages of 1% and 10%, respectively. Figure 11 shows the decrease of density by the raise of swelling capacity by 110% for the pure (silica-free) particles and Figure 12 shows an 80% increase in equilibrium swelling capacity of NS-10-0.5 compared with NS-10-2.

(13) $$R^{2}=\frac{{\left[\sum_{i=1}^{N}\left(O_{i}-\bar{O}\right)\cdot \left(P_{i}-\bar{P}\right)\right]}^{2}}{\sum_{i=1}^{N}\left(O_{i}-\bar{O}\right)\cdot \sum_{i=1}^{N}\left(P_{i}-\bar{P}\right)}\;$$

(14) $$r=\frac{N\left(\sum_{i=1}^{N}O_{i}\cdot P_{i}\right)-\left(\sum_{i=1}^{N}O_{i}\right)\cdot \left(\sum_{i=1}^{N}P_{i}\right)}{\sqrt{\left(N\sum_{i=1}^{N}O_{i}^{2}-{\left(\sum_{i=1}^{N}O_{i}\right)}^{2})\cdot (N\sum_{i=1}^{N}P_{i}^{2}-{\left(\sum_{i=1}^{N}P_{i}\right)}^{2}\right)}}\;$$

(15) $$RMSE=\sqrt{\sum_{i=1}^{N}\frac{1}{N}{\left(O_{i}-P_{i}\right)}^{2}}$$

$P_{i}=predicted\;values\;in\;sample\;i$

$O_{i}=observed\;values\;in\;sample\;i$

P=predicted values

O=observed values

$\bar{P}=predicted\;values$

$\bar{O}=mean\;of\;observed\;variables$

N=number of training or testing samples

### 3.2. Developing of ELM-ANFIS

**Figure 13:**
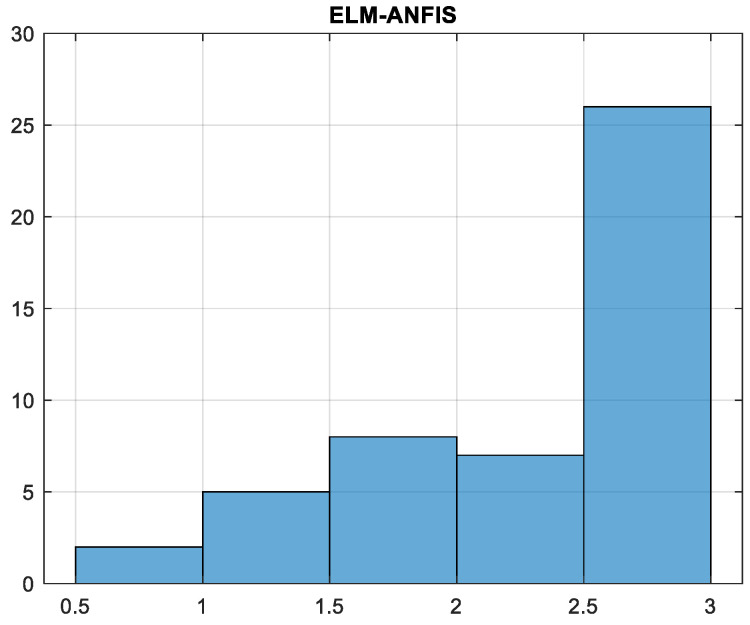
Error distribution of data.

**Figure 14:**
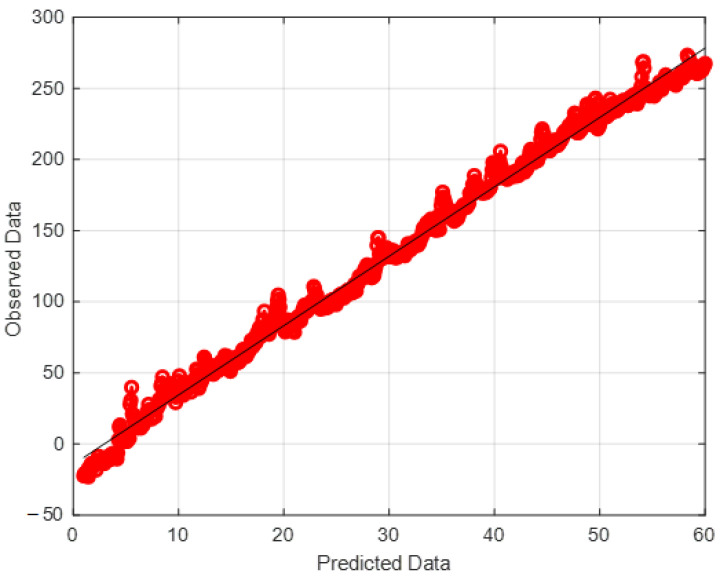
Distribution of data.

**Figure 15:**
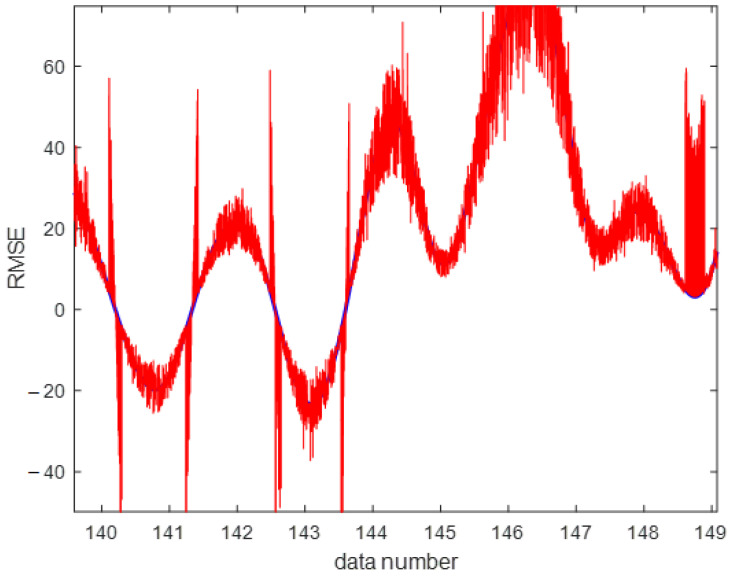
RMSE of the model.

**Figure 16:**
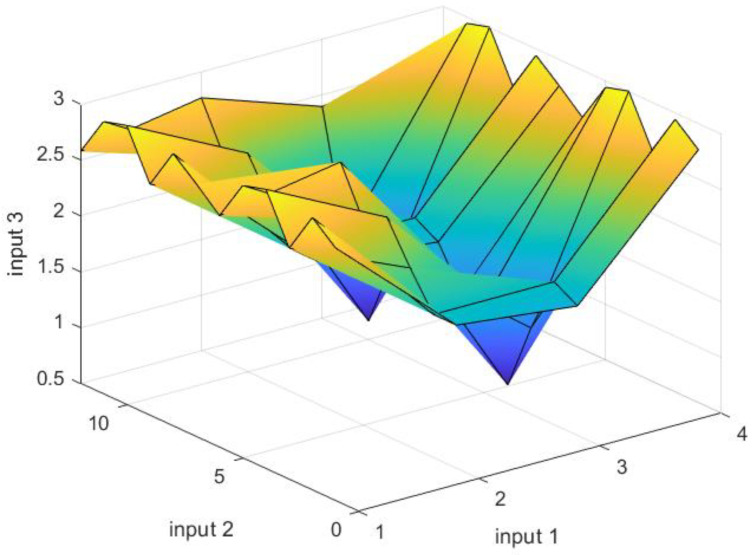
3D plot of ANFIS-ELM.

In the case of the regression analysis on real datasets, the ANFIS-ELM function was designed and modified accordingly. A total of 80 trials were carried out for each experiment with the findings being published after an average of 50 trials. There were 50 neurons found in this research, which was a significant number. According to ELM, the first number of nodes was determined by a process of hit-or-miss that was dependent on the quantity of data that was received at the time. Elimination of redundant or inactive hidden nodes allows ANFIS-ELM to provide more accuracy in terms of performance than before. The number of neurons was determined by the use of a trial-and-error procedure. Following that, the RMSE was used to validate the function of ELM throughout the training and testing phases. For instance, while using the classic ELM, the dataset of delta elevators with 3000 beginning nodes generated an RMSE of 0.6743; however, when using the ANFIS–ELM, the dataset of delta elevators with 3000 starting nodes produced an RMSE of 0.5987. ANFIS-ELM also takes much less time to train than a standard ELM, which saves time and cost for both phases. In terms of training time, ANFIS-ELM takes 0.1043 s and ELM takes 0.0231 s when the smallest dataset is used and a training size of 75 is used. For a conclusion, ANFIS-ELM reduces the likelihood of model overfitting. Figure 12 depicts the computed moment–rotation curves, which highlight the points at which the state of the system changes. This illustration demonstrates that the measured settlement is in good agreement with the forecast technique. Analyses of the data obtained by these procedures were used to estimate the distribution intervals of the data, which were then normalized in the range of 0 to 100 before being deformalized. In this particular case, the data distribution pattern was computed using the ELM-ANFIS software. The model’s histogram and error distribution are shown in Figure 13. The highest error occurred in the range of 2.5–3 with 25 data, while the least error occurred in the range of 0.5–1 with 2 data. The purpose of this research is to precisely assess the data produced from the methodologies in order to provide an analysis of replenishment of hydrogel void space concrete with nano-silica particles. The regression line and red dots in Figure 14 are the noises in this examination. The intensity of gray noises along the line indicates that our model is better at prediction. Any overlap between the line and the red dots indicates how close the predicted and observed values are aligned. Figure 15 depicts the RMSE test results, with RMSE errors ranging from −40 to 60. Overlaps seen between the predicted (red line) and observed (blue line) values indicate the proposed model’s accuracy. Figure 16 shows the 3D plot of ANFIS-ELM. There is a close overlap between two values in this Figure, which represents the model’s outperformance. Table 1 reveals that the *R^2^* of the test phase in ELM-ANFIS model is 0.8796 which is closer to 1. In one-layer testing, the RMSE (0.5987) also revealed a better outcome as it was close to zero. It is possible to acquire highly accurate findings for the prediction of intricate subsidence patterns induced by mining using the exact analysis given by this hybrid. As a result, ELM-ANFIS may be able to perform significantly better.

## 4. Conclusions

When comparing silica-containing polyacrylamide composite hydrogel particles to silica-free hydrogel particles, the silica-containing polyacrylamide composite hydrogel particles resulted in a higher level of hydration of internally cured cement paste. Composite hydrogels with a small crosslink density and a greater silica dosage showed the largest equilibrium-free swelling capabilities of all the hydrogels tested. The increased swelling was caused by the silica, which allowed for more water absorption as well as the lower crosslink density, which allowed the polymer molecules to move more freely. When comparing pastes that contain silica-free hydrogel particles and hydrogel-free pastes, the electrical resistivity and compressive strength of pastes that comprise of composite hydrogel particles with higher crosslink density as well as a higher dosage of silica increased significantly with increasing age that was also compatible with the rise in non-evaporable water content. At later ages, sizes of hydrogel-related void seemed to be more significant for controlling the compressive strength in comparison with the local microstructure refinement owing to void-filling, which was seen earlier. In spite of this, the data indicated that the drop in strength caused by larger void sizes can be, at the very least, partially offset by an increase in hydrated product in the voids, which was made possible by the addition of silica in the hydrogel particles. It should go without saying that the connection between the degree of void size, hydration, and void-filling activity will have an impact on prolonged strength and that this is a critical structure–property connection when choosing the SAPs for internal curing in the first place. Practically, the addition of silica to the hydrogel’s polymer network allows for the addition of more cementitious elements in a new way that does not have the same negative effects as nanoparticles.

## Figures and Tables

**Table 1:** Regression results of the models in test phase.

AI Models	*R* ^2^	*RMSE*	*r*
**ANFIS**	0.7865	0.8758	1.5643
**ELM**	0.6754	0.6743	0.7865
**ANFIS-ELM**	0.8796	0.5987	0.4687

## Data Availability

Not applicable.
